# HSP70 Is a Critical Regulator of HSP90 Inhibitor’s Effectiveness in Preventing HCl-Induced Chronic Lung Injury and Pulmonary Fibrosis

**DOI:** 10.3390/ijms25031920

**Published:** 2024-02-05

**Authors:** Ruben M. L. Colunga Biancatelli, Pavel A. Solopov, Tierney Day, Betsy Gregory, Michael Osei-nkansah, Christiana Dimitropoulou, John D. Catravas

**Affiliations:** 1 Frank Reidy Research Center for Bioelectrics, Old Dominion University, Norfolk, VA 23509, USA; psolopov@odu.edu (P.A.S.); tday@odu.edu (T.D.); bgregory@odu.edu (B.G.); cdimitro@odu.edu (C.D.); jcatrava@odu.edu (J.D.C.); 2 Division of Pulmonary and Critical Care, Department of Internal Medicine, Eastern Virginia Medical School, Norfolk, VA 23507, USA; 3 School of Medicine, Eastern Virginia Medical School, Norfolk, VA 23507, USA; 4 School of Medical Diagnostic & Translational Sciences, College of Health Sciences, Old Dominion University, Norfolk, VA 23509, USA

**Keywords:** chronic lung injury, pulmonary fibrosis, hydrochloric acid, heat shock proteins, HSP90 inhibitors, HSP70, TAS-116, gefitinib, geranylgeranyl acetone

## Abstract

Exposure to hydrochloric acid (HCl) can provoke acute and chronic lung injury. Because of its extensive production for industrial use, frequent accidental exposures occur, making HCl one of the top five chemicals causing inhalation injuries. There are no Food and Drug Administration (FDA)-approved treatments for HCl exposure. Heat shock protein 90 (HSP90) inhibitors modulate transforming growth factor-β (TGF-β) signaling and the development of chemical-induced pulmonary fibrosis. However, little is known on the role of Heat Shock Protein 70 (HSP70) during injury and treatment with HSP90 inhibitors. We hypothesized that administration of geranylgeranyl-acetone (GGA), an HSP70 inducer, or gefitinib (GFT), an HSP70 suppressant, alone or in combination with the HSP90 inhibitor, TAS-116, would improve or worsen, respectively, HCl-induced chronic lung injury in vivo and endothelial barrier dysfunction in vitro. GGA, alone, improved HCl-induced human lung microvascular endothelial cells (HLMVEC) barrier dysfunction and, in combination with TAS-116, improved the protective effect of TAS-116. In mice, GGA reduced HCl toxicity and while TAS-116 alone blocked HCl-induced chronic lung injury, co-administration with GGA, resulted in further improvement. Conversely, GFT potentiated HCl-induced barrier dysfunction and impaired the antidotal effects of TAS-116. We conclude that combined treatments with HSP90 inhibitors and HSP70 inducers may represent a novel therapeutic approach to manage HCl-induced chronic lung injury and pulmonary fibrosis.

## 1. Introduction

Hydrochloric acid (HCl) is one of the most common chemicals employed in industry, automotive plants, and oil and drilling facilities with an estimated traffic of 20 million tons per year. As a result, HCl is considered one of the top five chemicals causing injuries from accidental exposures, according to the Hazardous Substances Emergency Events Surveillance [1]. Acutely, HCl causes cough, chest pain, acute respiratory distress syndrome and, depending on dose and duration of exposure, even death [2]. After the initial inflammation, HCl elicits a persistent profibrotic response, mediated by transforming growth factor-β (TGF-β) that has been related clinically to chronic conditions such as reactive airway dysfunction syndrome (RADS) [3,4], asthma-like conditions [5,6] and pulmonary fibrosis [7,8]. There are no FDA-approved drugs to target the long-lasting complications of HCl exposure.

**Figure 1:**
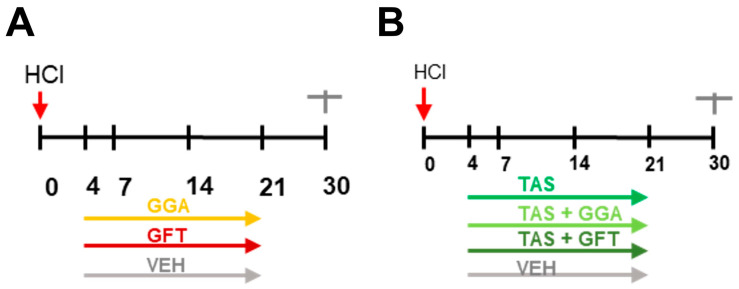
Schematic timelines of the in vivo studies. (**A**) After HCl instillation (*red arrows*), treatments began at day 4 with GGA (geranylgeranyl acetone 200 mg/kg 3×/week per os), GFT (Gefitinib 200 mg/kg 3×/week per os) or vehicle (10% DMSO in corn oil) till day 21. (**B**) After HCl instillation, treatments began at day 4 with TAS-116 (7 mg/kg 5×/week per os); TAS + GGA (7 mg/kg 5×/week per os plus 200 mg/kg 3×/week per os, respectively); TAS + GFT (7 mg/kg 5×/week per os plus 200 mg/kg 3×/week per os, respectively) or vehicle (10% DMSO in corn oil) till day 21. All groups were euthanized at day 30.

Heat shock proteins (HSPs) constitute a large group of chaperones that are active in response to thermal and other environmental stressors. HSPs are classified into HSPH (HSP110), HSPC (HSP90), HSPA (HSP70), DNAJ (HSP40), and HSPB (small HSP), with each of these families containing multiple genes, proteins, and isoforms [9]. We have previously showed that post-treatment with various inhibitors of heat shock protein 90 (HSP90, a member of the HSPC family) (AUY-922, AT13387, TAS-116) confers significant beneficial effects in preventing chemical (including HCl)-induced chronic lung injury and pulmonary fibrosis [10,11,12]. For TAS-116, we have defined the maximal delayed treatment start time (96 h after HCl), the minimal effective treatment dose (7 mg/kg, po, 5 days/week) and minimal period of treatment (three weeks) (Figure 1B) [13]. HSP90 is an ATP-dependent molecular chaperone that assists proteins in folding, stabilization, and if irreversibly damaged, degradation [14]. HSP90, together with HSP70 and other chaperones of the HSP family, constitute a sophisticated protein-quality-control network that guards the proteome during injury [15]. Furthermore, HSP90 plays a critical role in TGF-β signaling, as it stabilizes the TGF-β receptor, and chaperones both the canonical pathway, mediated by Smad proteins, and the non-canonical pathway, signaled by MAPK/ERK [16]. 

A common effect of HSP90 inhibitor treatment is the upregulation of HSP70 (a member of the HSPA superfamily). This mechanism is used as a biomarker of the biological efficacy of HSP90 inhibitors as well as of their cellular toxicity [17,18]. HSP70 acts either as an independent chaperone or, together with HSP90, as a co-chaperone [19]. It is associated with various cellular mechanisms with mainly protective functions [20,21]. For example, it blocks the function of protease activator factor 1 (PAF-1), the consequent protein kinase B (AKT) phosphorylation, and the B-cell Lymphoma 2 (Bcl-2) apoptotic pathway [22]; it impedes apoptotic pathways by interfering with caspase-3 and phospholipase A2 activation [23] and plays a critical role in the stress response by modulating the nuclear translocation of heat shock factor-1 (HSF-1). HSP70 is found in the cytoplasm, nucleus, and, minimally, in the extracellular space. In the cytoplasm, HSP70 is bound to HSF-1, but during stress, HSF-1 is released and translocated to the nucleus where it promotes the transcription of stress-induced genes encoding stress proteins necessary for cellular survival [24]. 

It is not clear if modulation of HSP70, in association with an HSP90 inhibitor, would result in an enhanced or impaired therapeutic profile [25]. Here, we have queried if the modulation of HSP70, by either HSP70 inhibitors or inducers, would affect HCl-induced lung toxicity, and if it would similarly influence the therapeutic profile of TAS-116 in preventing HCl-induced chronic lung injury in mice. C57BL/6j wild-type mice were intratracheally instilled with HCl and treated with TAS-116 alone or in combination with either the HSP70 inhibitor Gefitinib or the HSP70 inducer geranylgeranyl acetone, both at 200 mg/kg, per os 3×/week, as similarly employed by others [26]. At the end of the experimental period (30 days) molecular, histological, and functional outcomes were investigated. 

## 2. Results

### 2.1. Timeline of Animal Studies 

At day 0, mice were intratracheally instilled with 0.1 N HCl or saline, in volumes not exceeding 50 µL (2 µL/g) and monitored till euthanasia at day 30 post-instillation. From day 4 and till day 21, mice instilled with HCl received treatments with either GGA (200 mg/kg, 3×/week per os), GFT (200 mg/kg, 3×/week per os) or vehicle (10% DMSO in corn oil) (Figure 1A). In another set of experiments, HCl-instilled mice were treated with TAS-116 (7 mg/kg, 5×/week per os) administered alone or in combination with GGA (200 mg/kg, 3×/week per os), or GFT (200 mg/kg, 3×/week per os) (Figure 1B). Then, bronchoalveolar lavage fluid (BALF) and lung tissue were collected at day 30.

### 2.2. HSP70 Upregulation Improves HCl-Induced Lung Dysfunction

**Figure 2:**
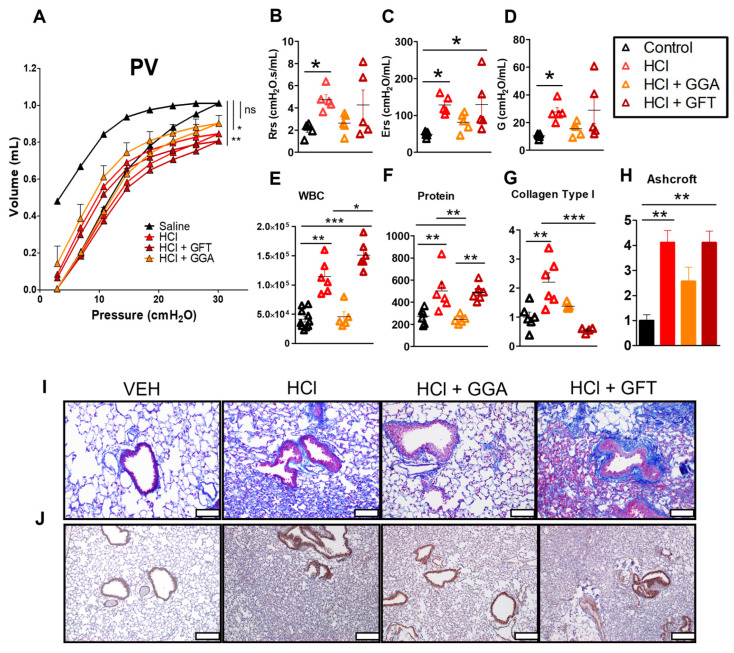
HSP70 upregulation improved lung function and reduced the development of lung fibrosis in response to hydrochloric acid (HCl). The Flexivent system was used to measure parameters of lung function including PV loops (**A**), Total respiratory resistance (Rrs), (**B**) elastance (Ers), (**C**) tissue damping (G) (**D**). BAL fluid (BALF) was analyzed for (**E**) WBC and (**F**) proteinosis. Collagen was measured in lung homogenates (**G**), representative images of Masson’s trichrome (**I**) and HSP70 staining (**J**) were collected, and Ashcroft score (**H**) was evaluated for all groups. Evaluation of the Ashcroft score was performed by blinded investigators. Scale white bars 100 µm. Mean ± SEM; n way ANOVA and Bonferroni’s post-test = 5–6; ns: not significant; *: *p* < 0.05; **: *p* < 0.01; ***: *p* < 0.001 with either 1-way ANOVA and Tukey’s or 2-way ANOVA and Bonferroni’s post-test.

Mice instilled with HCl and treated with vehicle displayed a downward shift in Pressure–Volume (PV) loops when compared to mice instilled with saline (Figure 2A), and an increase in total respiratory resistance (Rrs), elastance (Ers) and tissue damping (G) (Figure 2B–D). Mice treated with the HSP70 inducer, GGA, displayed an improvement in the HCl-induced downward shift of PV loops and values of Rrs, Ers and G. However, treatment with the HSP70 inhibitor, GFT did not significantly alter the effects of HCl on lung dysfunction.

### 2.3. HSP70 Upregulation Ameliorates HCl-Induced Persistent Inflammation and Lung Fibrosis

HCl exposure provoked persistent alveolar inflammation as indicated by the increase in white blood cells (WBC) and proteins in the BAL fluid at 30 days (Figure 2E,F). Lung sections stained with Masson’s trichrome also indicated that HCl induced septal thickening, peribronchial collagen deposition, and alveolar collapse in an overall picture of chronic lung injury as quantitated by the Ashcroft score (Figure 2H,I) and also by the increased values of collagen type I (Figure 2G). Mice instilled with HCl and treated with GGA exhibited significant reductions in inflammation and in the magnitude of fibrosis. The HSP70 inhibitor, GFT, worsened the alveolar inflammation (Figure 2E) but did not affect HCl-induced chronic lung injury. Lung sections immunostained for HSP70 demonstrated an increased expression in the HCl group alone (Figure 2J).

### 2.4. HCl Exposure Activates Heat Shock Protein 90

**Figure 3:**
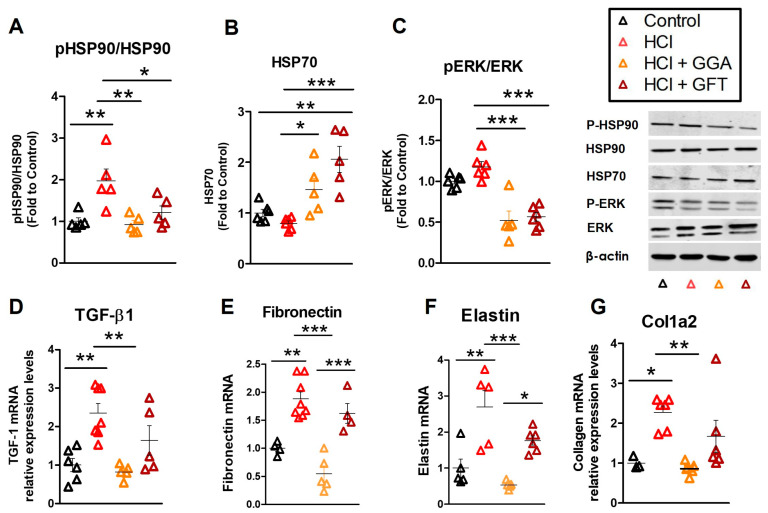
HSP70 upregulation improved activation of the HSP network, TGF-β1 signaling and ECM deposition induced by HCl. Lung tissue was collected 30 days after instillation with either HCl or saline. Treatments were as in Figure 1. Lung homogenates were analyzed by Western blotting for (**A**) phopsho-HSP90 and total HSP90, (**B**) HSP70, (**C**) phospho-ERK, and total ERK. Phosphorylated proteins were normalized to their respective total proteins, while other proteins were normalized to β-actin and then to the average of control (saline-instilled). RNA was extracted from TRIzol-isolated tissue and mRNA for (**D**) TGF-β1, (**E**) fibronectin, (**F**) elastin, and (**G**) collagen type 1α2 were quantitated and normalized to β-actin mRNA and then to the average of control mice (saline). Mean ± SEM; n = 5–6; *: *p* < 0.05; **: *p* < 0.01; ***: *p* < 0.001 with 1-way ANOVA and Tukey’s post-test.

Exposure of mice to HCl provokes persistent activation of HSP90, i.e., phosphorylation, in the lungs [10]. Similarly, inflamed cancerous tissues display an increase in HSP90 activation/phosphorylation. Since HSP90 inhibitors have a 1000-fold higher affinity for the phosphorylated form, doses that inhibit active HSP90, mostly spare inactive HSP90 in healthy tissues and thus provoke minimal side effects [27]. HCl elicited activation of HSP90 (Figure 3A), without influencing HSP70 levels (Figure 3B). HCl also promoted the expression of MAPK/ERK a common kinase involved in cellular signaling and the client protein of HSP90 (Figure 3C). Administration of either GGA or GFT modulated MAPK/ERK and, also, the phosphorylation of HSP90. GGA and GFT similarly increased the expression of HSP70 at 30 days after HCl exposure (Figure 3B). 

### 2.5. HSP70 Upregulation Modulates the HCl-Induced Profibrotic Response

TGF-β is the leading pro-fibrotic cytokine, responsible for disease progression and severity in humans [28]. HCl caused higher and persistent expression of transforming growth factor-β1 (TGF-β1) mRNA compared to mice instilled with saline (Figure 3D). Similar increases were observed in the mRNA of extracellular matrix proteins (ECM) fibronectin, elastin, and collagen type 1α2 (Figure 3E–G). HCl-instilled mice treated with GFT did not show any significant changes compared to HCl-instilled mice treated with vehicle, while those treated with GGA displayed reductions in the TGF-β axis and ECM proteins (Figure 3D–G). 

### 2.6. HSP70 Upregulation Improved the TAS-116 Therapeutic Profile for HCl-Induced Lung Dysfunction

**Figure 4:**
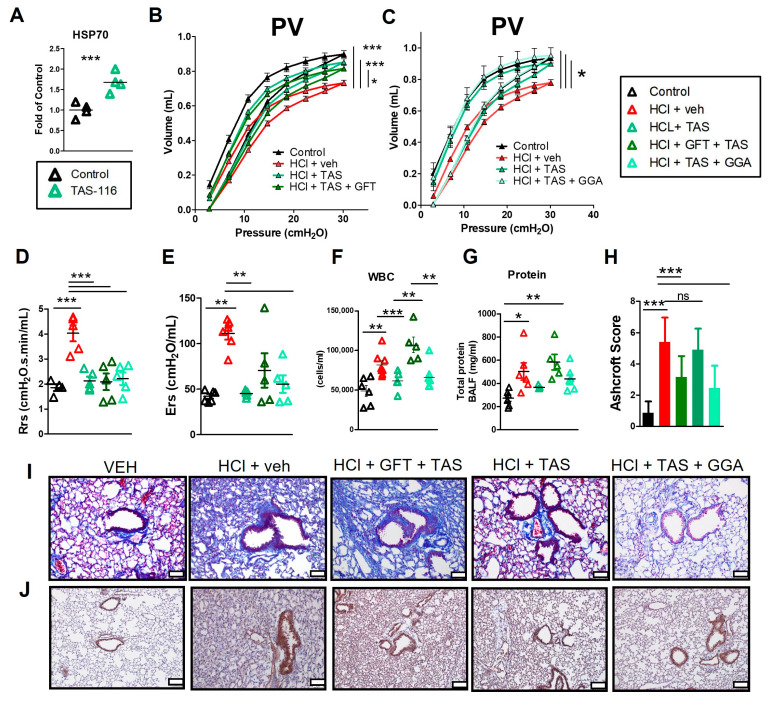
GFT impaired the effectiveness of the HSP90 inhibitor, TAS-116, in improving lung function and the development of pulmonary fibrosis following exposure to HCl. (**A**) HSP70 levels in mice treated for 17 days with 7 mg/kg of TAS-116 per os 5×/week. The Flexivent system was used to measure parameters of lung function including (**B**,**C**) PV loops, (**D**) total respiratory resistance (Rrs), and (**E**) elastance. BAL fluid (BALF) was analyzed for (**F**) WBC and (**G**) protein concentration. Representative images of Masson’s trichrome (**I**) and HSP70 staining (**J**) were collected, and Ashcroft score (**H**) was evaluated for all groups. Evaluation of the Ashcroft score was performed by blinded investigators. Scale white bars 100 µm. Mean ± SEM; n = 5–6; ns: not significant; *: *p* < 0.05; **: *p* < 0.01; ***: *p* < 0.001 with 1-way ANOVA and Tukey’s post hoc test.

Inhibition of HSP90 is characterized by consequent increase in HSP70 levels [18]. As shown in Figure 4A, treatment with 7 mg/kg TAS-116 5×/week per os for 14 days, resulted in increased expression of HSP70 in the lungs. Then, to investigate whether the treatment with HSP90 inhibitors would have been affected by simultaneous modulation of HSP70, HCl-instilled mice were treated with TAS-116 in combination with either GGA (HSP70 inducer) or GFT (HSP70 inhibitor). TAS-116, GGA and GFT were employed starting 4 days post HCl and till day 21. Tissue was then collected at day 30 (Figure 1B). Treatment with TAS-116 improved HCl-induced downward shift of PV loops, that was slightly worsened in the TAS + GFT group (Figure 4B). However, TAS-116 supplemented with GGA restored lung PV relationships to those of saline-instilled controls (Figure 4C). Treatments with TAS-116 or TAS-116 and GGA also improved total respiratory resistance (Rrs) and Elastance (Ers) (Figure 4D,E), but not treatment with TAS-116 combined with GFT.

### 2.7. GFT Impaired HSP90 Inhibitor Amelioration of HCl-Induced Persistent Inflammation and Lung Fibrosis

Treatments with TAS-116 improved both the number of leukocytes and the level of proteins in BALF (Figure 4F,G). TAS-116 further reduced the visible deposition of collagen, preserved lung architecture, and diminished the HCl-induced alveolar and septal thickening (Figure 4I). Treatment with TAS-116 plus GGA similarly prevented the HCl-induced alveolar inflammation and histological evidence of fibrosis, as reflected in the Ashcroft score (Figure 4H). GFT impaired the protective effects of TAS-116 resulting in a higher Ashcroft score and persistent proteinosis and cellularity, when compared to mice exposed to HCl and treated with TAS-116. Lung sections stained for HSP70 indicated higher expression only in the HCl plus vehicle group (Figure 4J).

### 2.8. TAS-116 Reduced HCl-Induced Activation of Heat Shock Protein 90

**Figure 5:**
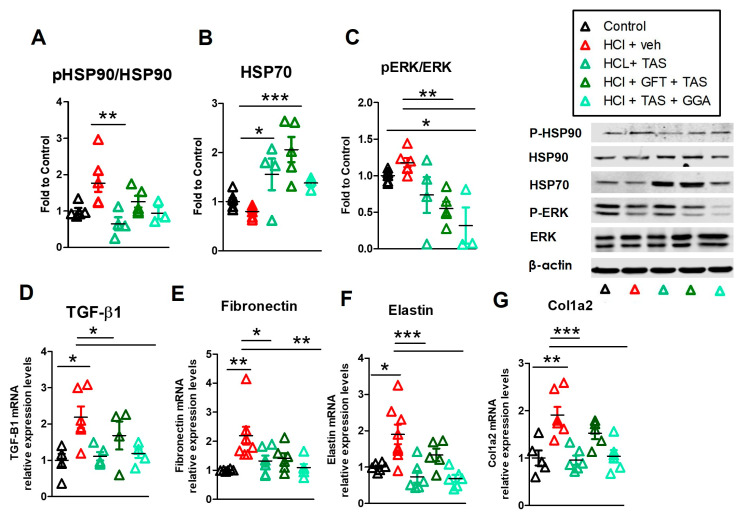
HSP70 inhibition impaired the effectiveness of the HSP90 inhibitor, TAS-116, to reduce HCl-induced HSP90 activation, TGF- β1 signaling and ECM deposition. Lung tissue was collected 30 days after instillation with either HCl or saline. HCl-instilled mice were treated as in Figure 1. Lung homogenates were analyzed by Western blotting for (**A**) phopsho-HSP90 and total HSP90, (**B**) HSP70, (**C**) phospho-ERK, and total ERK. Phosphorylated proteins were normalized to their respective total proteins, while other proteins were normalized to β-actin and then to the average of control (saline-instilled) values. RNA was extracted from TRIzol-isolated tissue and mRNA for (**D**) TGF-β1, (**E**) fibronectin, (**F**) elastin, (**G**) collagen type 1α2 were quantitated and normalized to β-actin mRNA and then to the average of control (saline instilled) values. Mean ± SEM; n = 5–6; *: *p* < 0.05; **: *p* < 0.01; ***: *p* < 0.001 with 1-way ANOVA and Tukey’s post hoc test.

Treatment with the HSP90 inhibitor TAS-116 blocked the activation (phosphorylation) of HSP90 and increased HSP70 levels (Figure 5A,B). Combined therapies with GGA of GFT, resulted in reduced p-HSP90 levels. HSP70 levels were upregulated in the TAS-116 group and Fluctuated in the TAS-116 + GFT and TAS + GGA groups. The MAPK/ERK signaling was dampened in all treatment groups (Figure 5C).

### 2.9. GGA Ameliorates the HCl-Induced Profibrotic Response

Treatments with TAS-116 alone or in combination with GGA resulted in reduction of HCl-induced activation of the TGF-β1 axis and consequent reduced production of ECM proteins such as fibronectin, elastin and collagen type 1α2 (Figure 5D). However, when therapy with HSP90 inhibitor TAS-116 was combined with the HSP70 inhibitor, GFT, a persistent activation of TGF-β1 and overproduction of elastin and collagen, was observed (Figure 5F,G).

### 2.10. HSP70 Upregulation Improves TAS-116 Therapeutic Effects on HCl-Induced Hyperpermeability

**Figure 6:**
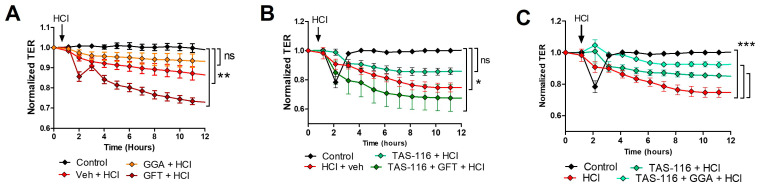
TAS-116 protective effect on HCl-induced loss of endothelial barrier integrity is dependent on HSP70. (**A**) Human lung microvascular endothelial cells (HLMVEC) were grown on gold electrodes till a stable resistance (>800 Ω) was achieved. They were pre-treated with vehicle, 20 µM geranylgeranyl acetone [25] or 20 µM gefitinib (GFT) for 12 h and then challenged with HCl (black arrows). (**B**) HLMVEC were pre-treated with TAS-116 (4 µM), or TAS + GGA (4 µM and 20 µM, respectively) for 12 h and were then exposed to HCl. (**C**) HLMVEC were pre-treated with TAS-116 (4 µM), or TAS + GFT (4 µM and 20 µM, respectively) for 12 h and were then exposed to HCl. Means ± SEM; n = 4 per group; ns: not significant; *: *p* < 0.05; **: *p* < 0.01; ***: *p* < 0.001 with 2-way ANOVA and Bonferroni’s post hoc test.

Previous reports suggest that HSP90 inhibitors exert a protective effect on lung endothelial barrier function [11,29]. Thus, we first investigated if modulation of HSP70 would affects the severity of HCl-induced hyperpermeability and then, if it would similarly modulate the protective effects of TAS-116. Cells seeded in 9610idf arrays were pre-treated for 12 h with either vehicle, GGA (20 µM) or GFT (20 µM) and then exposed to HCl (0.02 N). HCl evoked a time-dependent reduction in transendothelial electrical resistance (TEER) that was significantly improved by the HSP70 inducer, GGA but worsened by the HSP70 inhibitor, GFT (Figure 6A). Pretreatment with TAS-116 (4 µM) for 12 h prevented the loss of endothelial barrier function induced by HCl (Figure 6B). Combined treatment of TAS-116 with GFT impaired the protective effect of TAS-116 and displayed a worsen profile than TAS-116 alone. Conversely, cells pre-treated with TAS-116 + GGA and then exposed to HCl, demonstrated a minimal reduction in TER, compared to HCl-treated cells (Figure 6C).

## 3. Discussion

Pulmonary fibrosis (PF) is a devastating disease, with limited therapeutic interventions [30,31]. Current treatment options are focused on modulating TGF-β signaling and its deleterious effects on lung parenchyma [32,33]. Increased levels of TGF-β are responsible for the progressive epithelial to mesenchymal transformation, the activation of macrophages and fibroblasts and the increased deposition of extracellular matrix in the lung, reducing lung elasticity and airway function [34,35].

We have previously characterized time and dose responses to HCl instillation, and identified a first strong inflammatory response, that fades by day 4 post-instillation, followed by a lingering moderate inflammation and TGF-β activation that persist at least till day 30 [7]. We have then shown that treatment with TAS-116 starting 4 days post HCl exposure, prevented chronic lung injury by targeting the TGF-β profibrotic response, and not by modulating the initial inflammation [13].

In this study, we have investigated the role of HSP70 during HCl-induced toxicity and treatment with the HSP90 inhibitor TAS-116. Previous studies have shown that treatment with recombinant HSP70 blocked apoptosis and prevented sepsis-induced lung injury [36]. Similarly, HSP70 overexpression inhibited caspase 3, 8, 9, APAF-1 and Bcl2 activation, preventing Acute Respiratory Distress Syndrome (ARDS) induced by cecal ligation and puncture in rats [37]. In our study, treatment with GGA, upregulated HSP70 and reduced the development of lung dysfunction and the histological abnormalities observed in HCl-exposed mice. Conversely, inhibition of HSP70, by GFT did not significantly affect the severity of HCl-induced pulmonary fibrosis (Figure 2 and Figure 3).

Increased HSP90 levels have been detected in plasma and in lung tissue of patients with pulmonary fibrosis [38,39]. This is the result of increased protein quality control necessary to assist cells during the cellular signaling and production of extracellular matrix proteins observed in pulmonary fibrosis [40]. Indeed, HSP90 binds extracellular fibronectin through its N-terminal fragment, thus regulating its expression [41]. HSP90 is similarly involved in promoting TGF-β signaling via direct stabilization of the TGFβ receptor [42], assistance to ERK, via CDC37, and regulating the nuclear translocation of Smad proteins [43,44]. For these reasons, multiple investigators have hypothesized that treatment with HSP90 inhibitors may represent a novel strategy for the management of pulmonary fibrosis [25,45,46]. However, it is not clear if this protective effect is mediated by the upregulation of HSP70, a common mechanism observed during HSP90 inhibition [18], and more importantly, if the association of HSP70 inducers with HSP90 inhibitors may result in an enhanced beneficial effect.

Thus, we treated HCl-instilled animals with TAS-116 alone or in combination with the HSP70 inducer, GGA or with the HSP70 inhibitor, GFT. Drugs were administered 4 days after HCl exposure, so that mice were treated beyond the initial powerful inflammatory response and only during the pro-fibrotic phase. Treatments with TAS-116 alone or in combination with GGA successfully prevented the HCl-induced downward shift in PV loops and improved the HCl-induced increase in resistance and in elastance. Both approaches also improved the lingering alveolar inflammation and as well as the histological evidence of fibrosis (Figure 4). This was further explained by the modulation of the TGF-β1 axis, reduced deposition of ECM proteins fibronectin, elastin and collagen and amelioration of the HSP network (Figure 5). TAS-116 and GGA further improved the endothelial barrier dysfunction induced by HCl, as indicated in Figure 6. Conversely, the HSP70 inhibitor gefitinib impaired the protective effects of HSP90 inhibitors. 

One of the novel findings of this study is that simultaneous administration of an HSP70 inducer together with an HSP90 inhibitor resulted in an improved therapeutic profile compared to HSP90 inhibitors alone in preventing HCl-induced chronic lung injury and pulmonary fibrosis. 

HSP90 inhibitors are currently investigated for cancer, cardiovascular and inflammatory diseases (clinicaltrials.gov, accessed on 20 December 2023) [47]. In cancer, the combination of HSP90 and HSP70 inhibitors exhibits enhanced therapeutic profile [48]. The purpose of this combined therapy is, to block the HSP response, prevent the cytoprotective effects of HSP70 and promote cell death and anti-proliferative mechanisms [49]. 

**Figure 7:**
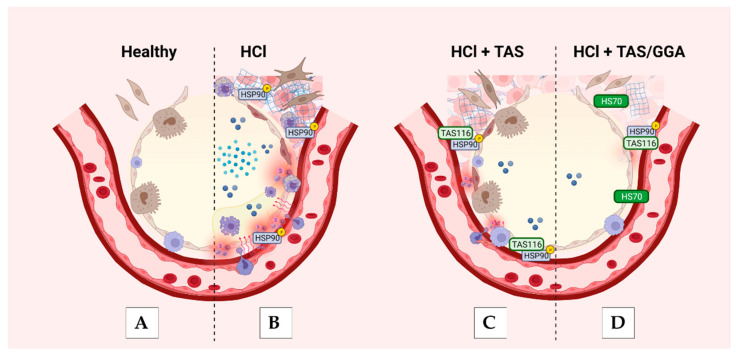
Proposed protective mechanisms of TAS-116 and TAS-116 + GGA against HCl induced chronic injury to lung alveolo-capillary structures. (**A**) Healthy alveolus with alveolar type I cells, alveolar type II (*cuboidal*) and resident macrophages. (**B**) During HCl exposure, injury to the endothelial barrier function with migration of neutrophils and monocyte-macrophages, there is fluid leak in the alveolar space, production of inflammatory cytokines, necrosis of alveolar epithelial cells, type I, with infiltration of myo-fibroblast and deposition of extracellular matrix, guided by activation (phosphorylation, yellow “P” within the figure) of HSP90. (**C**) TAS-116 the HSP90 inhibitor, blocks HSP90 activity, preventing endothelial barrier dysfunction, alveolar proteinosis and infiltration of immune cells and fibroblasts, ultimately reducing the deposition of extracellular matrix proteins. (**D**) When TAS-116 is associated with GGA an overexpression of HSP70 is observed, that prevents the apoptotic mechanisms in alveolar epithelial cells, type I. Figure created with Biorender.com (DB257QEA08).

Differently, in pulmonary fibrosis and inflammation, a much lower dose of HSP90 inhibitors (than for cancer) is used in order to prevent HSP90 activation and TGF-signaling without affecting cell survival. Apoptosis of the alveolar-epithelial layer has been deeply implicated in the pathophysiology of chronic lung injury [50,51]. Thus, the impaired efficacy of HSP90 inhibitors when associated with the HSP70 inhibitor, Gefitinib, may have been related to increased apoptosis in the bronchial and alveolar epithelium, contrasting the palliative effects on TGF-β signaling. Conversely, when GGA was administered in combination with TAS-116, an anti-apoptotic effect may have been exerted on the lower airways, further protecting lungs structures from HCl (Figure 7). Thus, to investigate this hypothesis, we have performed TUNEL assays with unfortunately inconclusive results. This does not confute fully our hypothesis as samples are collected uniquely at 30 days from HCl exposure (9 days from the last GGA or GFT treatments), when the active apoptotic mechanisms may have exhausted into a chronic pathology and hyperactivation of TGF-β. 

The study has some limitations. We have investigated only one dose of GGA and GFT, even though literature reports indicate that at these doses these compounds effectively upregulate and inhibit HSP70, respectively. Furthermore, since treatment with TAS, GFT, and GGA was terminated nine days before tissue harvest, analysis of HSP70 levels at day 30 does not reflect the levels expected during the profibrotic period, the 17 days of treatment. Instead, the measured levels likely reflect feedback or rebound signaling mechanisms that occurred between day 22 and 30. 

## 4. Materials and Methods

### 4.1. Materials

Pimitespib (TAS-116) was obtained from MedChemExpress (Monmouth Junction, NJ, USA); HCl, ACS grade, corn oil, methacholine USP grade, RIPA buffer, and protease inhibitor cocktail were obtained from Sigma-Aldrich Corporation (St. Louis, MO, USA). Socumb (pentobarbital) USP grade, Anased (xylazine) USP grade, and Ketaset (ketamine) USP grade were supplied by Henry Schein Animal Health (Pittsburgh, PA, USA). Geranylgeranyl acetone and Gefitinib were obtained from TCI America (Portland, OR, USA). Formaldehyde (10%) was purchased from Thermo Fisher Scientific (Waltham, MA, USA), the BCA Protein assay kit from Pierce Co. (Rockford, IL, USA), EDTA and Western blot membranes from GE Healthcare (Chicago, IL, USA), TRIzol and SuperScript VILO reverse transcriptase kit from Invitrogen (Carlsbad, CA, USA), RNeasy Mini Kit from Qiagen (Hilden, Germany), and SYBR Green Master Mix from Applied Biosystems (Carlsbad, CA, USA). All primers used for real-time quantitative PCR were purchased from Integrated DNA Technologies, Inc. (Coralville, IA, USA). For SDS-PAGE, ProtoGel (30% acrylamide mix) and TEMED were from National Diagnostics (Atlanta, GA, USA), Tris–HCl buffer from Teknova (Hollister, CA, USA), 10% SDS and ammonium persulfate from Thermo Fisher Scientific, and Protein Dual Color Standards and Tricine Sample Buffer from Bio-Rad Laboratories (Hercules, CA, USA). All the antibodies were purchased from a reputable commercial source and have published immunospecificity data. For antibodies used in Western blotting, Hsp70 (#4872S), Erk (#4695S) and p-Erk (#9101S) were purchased from Cell Signaling Technology Inc. (Danvers, MA, USA); Hsp90 (#ab59459) from Abcam (Cambridge, MA, USA); β-actin (#A5441) from Sigma-Aldrich Corporation (St. Louis, MO, USA); p-Hsp90 (#PA5-105480) and Collagen1α2 (#PA5-50938) from Invitrogen (Carlsbad, CA, USA). Secondary IRDye 800CW goat anti-rabbit (926-32211) and IRDye 680RD goat anti-mouse (926-68070) were obtained from LI-COR Biosciences (Lincoln, NE, USA). 

### 4.2. Animal Procedures

Adult male C57Bl/6J mice (Jackson Laboratories, Bar Harbor, ME, USA; 8–10 weeks old) were housed under pathogen free conditions with food/water ad libitum. On day 0, mice were anesthetized by i.p. injection of ketamine (Ketaset 60 mg/kg) and xylazine (Anased 6 mg/kg). A pre-emptive bolus of saline was administered i.p. (10 µL/g) and mice were intratracheally instilled with HCl or saline [7]. Mice were immobilized on a disinfected surgical field and a small neck incision was made (~1 cm), salivary glands separated to visualize the trachea, and a fine, 20–25 G, catheter inserted through the mouth (~1.5 cm), in such a way that it could be seen through the walls of the trachea. This technique allows a visual confirmation of the tracheal insertion of the catheter, providing 100% success without mortality. Mice were then suspended vertically and freshly prepared HCl (0.1 N) or saline (2 µL/g) was intratracheally instilled followed by a bolus of air (100 µL) to push the solution towards to the lower airways. The catheter was then withdrawn, the neck incision was closed with Vetbond and mice were allowed time to recover in an oxygen chamber (5 L/min and slowly weaned to room air). After 4 h, mice were returned to their cages and monitored daily. To address the impact of HSP70 regulation alone or during treatment with HSP90 inhibitors we employed the HSP70 inducer geranylgeranyl-acetone (GGA; 200 mg/kg, 3×/week, per os) or the HSP70 inhibitor, Gefitinib (GFT; 200 mg/kg, 3×/week, per os). We chose the HSP90 inhibitor TAS-116 (7 mg/kg, per os, 5×/week) for its high efficacy and bioavailability when administered orally, as previously shown [13]. Treatments for all three drugs alone or in combination started 96 h after HCl instillation and lasted three weeks (Figure 1A,B). The following groups were investigated and euthanized at 30 days post HCl: (1) saline i.t.; (2) HCl i.t. and treated with vehicle (10% DMSO in corn oil); (3) HCl i.t. and treated with TAS-116; (4) HCl i.t. and treated with GGA; (5) HCl i.t. and treated with TAS-116 and GGA; (6) HCl i.t. and treated with TAS-116 and GFT. On day 30, bronchoalveolar lavage fluid (BALF) was collected, lung function measurements were made (Flexivent, SCIREQ, Montreal, QC, Canada)), lung tissue was harvested for Western Blotting or RT-PCR analysis and lungs were fixed for histology. Separate sets of mice were employed for histology, lung function and BALF/tissue harvest. All animal studies were approved by the Animal Care and Use Committee (IACUC) of the Old Dominion University.

### 4.3. Bronchoalveolar Lavage Fluid (BALF) Analysis

BALF was obtained by flushing lungs with 1 mL PBS. The fluid was centrifuged at 2500× *g* for 10 min, the supernatant stored at −80 °C for further total protein analysis, while the pellet was re-suspended in 1 mL PBS and white blood cells (WBC) determined using a hemocytometer.

### 4.4. Histopathology

At the end of the experimental period, animals were euthanized, a small transverse incision was made in the middle of the trachea, and lungs were instilled and inflated with 10% formaldehyde solution to a pressure of 15 cm H_2_O through a 20 G catheter. The trachea was then ligated, and lungs were removed from the thorax and placed in 10% formaldehyde solution for 72 h before processing. Mid-transverse slices were made from the formalin-fixed lungs and were embedded in paraffin. Sections (5 µm thick) were prepared from the blocks and stained with Masson’s trichrome stain and for HSP70. The HSP70 antibody (AB2865351; Thermofisher, MA, USA) was used at a dilution of 1:1280 and with a HRP conjugated secondary antibody. 10 slides were stained, and 10 slides were used as negative controls where the primary antibody was omitted. Positive control tissue (mouse esophagus) stained positive. Ten randomly selected fields from each slide were examined under 10× and 20× magnification. All of the Masson’s trichrome stained slides were scored according to the Ashcroft score in order to estimate the severity of pulmonary fibrosis [52]. The observer was blinded to the treatment. 

### 4.5. Tissue Collection

Immediately after euthanasia, the chest was opened, blood was collected from the heart through the right ventricle, and the pulmonary circulation was flushed out with sterile PBS containing EDTA injected into the right ventricle. Lungs were dissected from the thorax, snap-frozen in liquid nitrogen, and kept at −80 °C for subsequent processing and analysis.

### 4.6. Western Blot Analysis

Proteins in lung tissue homogenates were extracted from frozen lungs by sonication (50% amplitude, three times for 10 s) in ice-cold RIPA buffer with added protease inhibitor cocktail (100:1). Protein lysates were gently mixed for 3 h at 4 °C, centrifuged twice at 14,000× *g* for 10 min. Equal amounts of protein, were mixed with Tricine Sample Buffer 1:1, boiled for 5 min, and then separated on a 10–12% polyacrylamide SDS gel by electrophoresis, transferred to a nitrocellulose membrane, incubated with the appropriate primary antibody, followed by incubation with the secondary antibody, and then, detected by digital fluorescence imaging (LI-COR Odyssey CLx, Dallas, TX, USA). Beta-actin was used as the loading control. ImageJ software v.1.8.0 was used to perform densitometric quantification of the bands (http://imagej.nih.gov/ij; National Institutes of Health, Bethesda, MD, USA, accessed on 13 November 2023). Membranes were stripped in a stripping buffer for 20 min, blocked, and incubated with other primary and secondary antibodies.

### 4.7. Real-Time PCR (qPCR)

Lung tissue, stored in an RNAlater solution, was dried and homogenized in TRIzol^®^, followed by a cleaning step using the RNeasy Mini Kit. The purified RNA was transcribed into cDNA using the SuperScriptTM IV VILO Reverse transcriptase Kit and was analyzed by real-time qPCR with SYBR Green Master Mix on a StepOne Plus Real-Time PCR System (Applied Biosystems v.2.3, Waltham, MA, USA). The results were evaluated using the standard curve method and were expressed as the fold of the control values, normalized to β-actin. Specifically designed primer pairs and qPCR conditions were applied to selectively determine the expression of mouse β-actin, fibronectin, collagen 1α2 and elastin. Fibronectin, elastin, collagen 1α2 and β-actin primers were as follows: Fibronectin F: GAA GTC GCA AGG AAA CAA GC, Fibronectin R: GTT GTA GGT GAA CGG GAG GA; elastin F: GGA GTT CCC GGT GGA GTC TAT T, elastin R: ACC AGG AAT GCC ACC AAC ACC TG; Collagen 1α2 F: GAA GCA CGT CTG GTT TGG A, Collagen 1α2 R: ACT CGA ACG GGA ATC CAT C; Beta actin F: 50-CCC CTG AGG AGC ACC GTG TG -30; Beta-actin R: 50-ATG GCT GGG GTG TTG AAG GT-30.

### 4.8. Lung Function Measurements

Mice were anesthetized with pentobarbital (120 mg/kg, i.p.), tracheostomized, and connected to a FlexiVent small animal ventilator (SCIREQ Inc., Montreal, QC, Canada), as previously published [28]. Ventilation was performed at a tidal volume of 10 mL/kg and respiratory rate of 150/min. A 15 min stabilization period was allowed before any measurements began. Firstly, following two total lung capacity maneuvers, pressure-volume loops were recorded three times. Secondly, Snapshot-150 and Quick Prime-3 maneuvers were performed to record respiratory system resistance (Rrs) and elastance [1], Newtonian resistance (Rn); tissue damping (G); inspiratory capacity (A); and the curvature of the PV loops (K) and presented as a mean of 12 recordings. Then, increasing doses of methacholine (0–50 mg/mL) were used to study bronchial reactivity, computing averages of 8 measurements per dose.

### 4.9. Endothelial Barrier Function

In-house harvested human lung microvascular endothelial cells (HLMVEC) were maintained in M199 media supplemented with 20% FBS and antibiotics/antimycotics, as described previously [29]. HLMVEC were cultured in 100 mm dishes until 90–95% confluency. HLMVEC were then seeded on gold electrode arrays (96w10idf), and endothelial barrier integrity was estimated by the electric cell-substrate impedance sensing (ECIS) technique, using an ECIS-ζθ instrument (Applied BioPhysics, Troy, NY, USA). Experiments were conducted when a stable resistance was reached and maintained above 800 Ω, as we have previously published [30]. Cells were incubated with 20 µM geranylgeranyl acetone (GGA), 20 µM gefitinib (GFT) or vehicle (10% DMSO), alone, or with the combined treatments of GGA + TAS-116 (20 µM and 4 µM, respectively) or GFT + TAS-116 (20 µM and 4 µM, respectively). After 12 h, cells were exposed to HCl (0.02 N). Changes in TER were continuously recorded. Experiments were performed in triplicates and repeated at least three times. Resistance values were collected and normalized to each well’s value at t = 0. Data are presented as means ± SEM.

### 4.10. Statistical Analysis

Statistical analysis was performed with GraphPad Prism Software (version 5.04, GraphPad Software, San Diego, CA, USA, accessed on 9 December 2023) and significance was set for *p* < 0.05 determined by either 1- or 2-way analysis of variance (ANOVA), followed by Tukey’s or Bonferroni’s post hoc test.

## 5. Conclusions

Combined therapies with HSP70 inducers may represent a novel strategy to boost the efficacy of HSP90 inhibitors for chemical-induced chronic lung injury and pulmonary fibrosis. Further studies are necessary to define optimal dose strategies, toxicity, and efficacy across different animals’ species.

## Data Availability

The data presented in this study are available on request from the corresponding author.
